# Does a digital support intervention reduce sedentary behaviour during cancer treatment in individuals with breast, prostate or colorectal cancer? Study protocol for a Swedish multicentre randomised controlled trial

**DOI:** 10.1136/bmjopen-2026-125120

**Published:** 2026-10-29

**Authors:** Lisa Rådman, Johanna Zetterlund, Petra von Heideken Wågert, Anna Henriksson

**Affiliations:** 1 Department of Health Sciences, Innovation and Design, Mälardalen University, Västerås, Sweden; 2 Centre for Welfare Change, Department of Health Sciences, Innovation and Design, Mälardalen University, Västerås, Sweden; 3 Department of Women’s and Children’s Health, Uppsala university, Uppsala, Sweden

**Keywords:** ONCOLOGY, Clinical Trial, Behavior, Digital Technology, eHealth, Physical Therapy Modalities

## Abstract

**Introduction:**

Sedentary behaviour increases during cancer treatment, often because of side effects from treatment. However, evidence on interventions specifically targeting sedentary behaviour during active cancer treatment is limited. Therefore, the aims of the reduce sedentary behaviour during cancer treatment (RedSedCan) study are to investigate the effects of sedentary behaviour on health outcomes and to explore the economic, environmental and social sustainability perspectives of a digital support intervention for reducing sedentary behaviour in individuals with breast, prostate or colorectal cancer receiving neoadjuvant or adjuvant cancer treatment. In addition, participants’ experiences with the intervention as well as healthcare professionals’ perspectives for supporting individuals with cancer to reduce sedentary behaviour will be explored.

**Methods and analysis:**

A multicentre randomised controlled trial will be conducted with short-term (3 months) and long-term (6, 12 and 24 months) follow-up. After baseline assessments, participants will be randomised into one of two groups as follows: a digital support intervention group or a control group receiving usual care. The digital support intervention—the RedSedCan intervention—consists of access to a home page containing information and tips about how to reduce sedentary behaviour, a commercially available step-counting mobile application, use of mobile phone reminder/alarm functions and four digital meetings with physiotherapists. During these meetings, participants will receive support for setting and reviewing goals. The main outcome is time spent engaging in sedentary behaviour, which is measured with a thigh-worn accelerometer at 3 months. Secondary outcomes include long-term follow-ups, physical activity, health-related outcomes, health economic effects and greenhouse gas emissions from interventions and healthcare. In addition, the experiences of participants undergoing the intervention and the attitudes and perceived barriers and facilitating factors of healthcare professionals for supporting individuals with cancer to reduce sedentary behaviour will be explored qualitatively.

**Ethics and dissemination:**

Ethical approval has been obtained from the appropriate ethics review authority. Findings will be disseminated through peer-reviewed publications, conference presentations and other relevant scientific and patient communities.

**Trial registration number:**

NCT06719206.

**STRENGTHS AND LIMITATIONS OF THIS STUDY**

- This multicentre randomised controlled trial uses both short- and long-term follow-up (up to 24 months), enabling evaluation of the intervention effects over time.
- Sedentary time, the primary outcome, is measured objectively using a thigh-worn accelerometer, reducing the risk of self-report bias.
- The study integrates quantitative, qualitative, health economic and environmental sustainability evaluations to provide a comprehensive assessment of the intervention and its implementation.
- The digital intervention is designed to be scalable and accessible for individuals undergoing cancer treatment across different cancer diagnoses and treatment settings.
- Blinding of participants and treating physiotherapists is not feasible because of the nature of the behavioural intervention.

## Introduction

 Physical activity plays a crucial role in promoting health during and after cancer treatment. Regular physical activity helps reduce treatment-related side effects, maintain functional capacity, enhance health-related quality of life and lower mortality risk.^1^ To reduce the risk of secondary diseases and premature mortality, at least 60 min of physical activity per day is recommended during cancer treatment, in contrast to 150–300 min of moderate-intensity or 75–150 min of vigorous-intensity activity per week for healthy adults.^2^ Beyond physical activity levels, sedentary behaviour (ie, any waking time spent sitting or lying with an energy expenditure below 1.5 metabolic equivalents)^3^ poses independent health risks. Current guidelines for healthy individuals recommend limiting sitting or lying time during the day^4^; however, adhering to both physical activity recommendations and sedentary time is particularly challenging among individuals with chronic conditions or treatment-related fatigue.

Receiving a cancer diagnosis is a significant source of distress and anxiety.^5^ Treatments can be demanding and commonly include chemotherapy, radiation therapy, hormonal therapy or targeted biological therapies. These treatments can disrupt daily routines and participation in daily activities because of various side effects, such as cancer-related fatigue, pain, nausea, infections or depression.^6^ Fatigue and depression have been identified as major barriers to maintaining physical activity among patients with cancer.^7^ Consequently, owing to these side effects, many patients decrease their physical activity levels while increasing their level of sedentary behaviour during treatment.^8^ One epidemiological study has reported an average sedentary time of 9.5 hours per day in patients with breast and colorectal cancer, with only 26 min per day spent in moderate-to-vigorous physical activity.^9^ Longer uninterrupted sedentary bouts (>20 min) have been associated with lower quality of life in patients with breast cancer,^10^ and similar patterns have been observed in patients with colorectal cancer.^11^

The elevated risk of additional health complications, such as noncommunicable diseases, for example, mental health issues, type 2 diabetes^6^ and cardiovascular disease,^12^ can be partly attributed to physical inactivity and treatment side effects. Although high physical activity levels mitigate some negative effects of sedentary behaviour, they do not fully compensate for the harm associated with prolonged sedentary time, underscoring the need for interventions targeting sedentary behaviour.^13–15^ Prolonged sedentary time adversely affects multiple physiological systems, including glucose and lipid metabolism, vascular function and systemic inflammation, which may contribute to various chronic diseases such as type 2 diabetes, cardiovascular disease and certain cancers.^15 16^ Epidemiological studies have consistently linked higher sedentary behaviour levels to increased disease incidence and mortality. Sitting for more than 4 hours daily is associated with up to a 50% increased risk of all-cause mortality^13^ and a 12% increased risk of cancer incidence and mortality according to a meta-analysis.^17^ Both the total amount of sedentary behaviour and how it is accumulated are important. Longer uninterrupted sedentary bouts are associated with adverse cardiometabolic outcomes, independent of total sedentary time or physical activity levels.^13^ A recent meta-analysis has further suggested that relatively small reductions in sedentary behaviour may have meaningful population-level effects.^18^

However, in cancer populations, sedentary behaviour has been studied less extensively than physical activity and is often included only as a secondary outcome in intervention studies. Our research team previously explored the experiences of cancer patients in a phenomenological study and reported that sedentary behaviour may function as an adjustment to cancer treatment side effects. Sedentary behaviour is also influenced by other factors, such as work status and social support. Furthermore, patients tend to equate sedentary behaviour with being physically active or not, suggesting that the concept of sedentary behaviour is difficult to distinguish.^19^

Reducing sedentary behaviour provides independent health benefits, especially for individuals limited in physical activity due to illness, highlighting the relevance of person-centred approaches grounded in individual needs. A recent qualitative interview study exploring the opinions and preferences of patients for support to reduce sedentary behaviour during cancer treatment (RedSedCan) has highlighted the importance of healthcare professionals providing timely, repeated and individually adapted information.^20^ These findings highlight the importance of incorporating qualitative methods into intervention studies to better understand the needs and perspectives of patients.

Sedentary behaviour is prevalent during cancer treatment and it is a complex behaviour influenced by multiple factors, necessitating multifaceted behaviour change interventions. Evidence suggests that targeting sedentary behaviour reduction, rather than solely increasing physical activity, results in greater decreases in sedentary time.^21^ This underscores the need for interventions that target sedentary behaviour rather than relying solely on increasing physical activity.

To effectively reduce sedentary behaviour, interventions should include behaviour change techniques (BCTs).^22–24^ Previous research has identified goal setting, action planning, social support, graded tasks, self-monitoring, feedback on behaviour and prompts/cues as potentially effective for increasing physical activity among cancer survivors.^23 25^ Behaviour change interventions grounded in social cognitive theory may further strengthen these strategies, providing information, offering social support, delivering feedback, facilitating self-monitoring and establishing outcome goals.^26^ In addition, digital support solutions can facilitate adherence through reminders and goal-setting prompts. Implementing such technology in healthcare may offer a scalable, sustainable and potentially cost-effective approach to reduce sedentary behaviour and improve health outcomes. However, beyond assessing intervention effectiveness, understanding how and why such interventions work or not, and for whom, is essential—particularly for complex, digital and person-centred interventions. To achieve this goal, intervention studies increasingly incorporate quantitative and qualitative data alongside outcome evaluations to illuminate potential mechanisms of impact, contextual factors and perceived relevance. Quantitative data on participants’ engagement with and perceptions of an intervention can thus complement outcome-focused analyses, whereas qualitative methods can explore individuals’ experiences and contextual influences on behaviour change. Together, such approaches support the interpretation of intervention outcomes, contribute to a deeper understanding of underlying mechanisms and inform future refinement, implementation and sustainability. This is consistent with current recommendations for complex intervention research, which highlight the importance of understanding how interventions work, in what contexts and for whom, to support future implementation and sustainability.^27^

In addition to their potential to change behaviour, interventions targeting sedentary behaviour may have broader health, economic and sustainability effects. Both physical inactivity and sedentary behaviour are growing risk factors for poor health and, in relation to disease, require consideration of sustainability dimensions, including social, ecological and economic factors, in alignment with the United Nations Sustainable Development Goals.^28^ Oncological treatments often lead to side effects causing high healthcare utilisation and costs, and may reduce the ability of patients to return to work post-treatment.^18^ For example, a 12-week individualised exercise programme in women with early-stage breast cancer reduces healthcare utilisation during treatment.^29^ Despite evidence of high healthcare costs associated with sedentary behaviour, few studies have assessed the economic impact of sedentary behaviour reduction interventions in cancer survivors. However, a systematic review of five studies targeting workplace interventions to reduce sedentary time has revealed consistent cost-effectiveness.^30^ Reducing sedentary behaviour and physical inactivity during cancer treatment could thus decrease side effects and yield further health economic benefits. While exercise interventions have shown mixed cost-effectiveness results in cancer populations,^31^ digital solutions may offer more affordable alternatives and warrant further investigation.^32^ Evaluating all dimensions of sustainability (social, economic and environmental) alongside effectiveness outcomes is therefore important to capture the broader impact of interventions and to inform decision making regarding their long-term use and value in healthcare systems.

From an environmental sustainability perspective, healthcare delivery plays an important role in climate change mitigation, as anthropogenic greenhouse gas (GHG) emissions pose a growing threat to human health worldwide.^33^ Healthcare delivery contributes substantially to GHG emissions via medical care, pharmaceutical production and patient transportation.^34^ In Sweden, healthcare accounts for approximately 21% of public GHG emissions, equating to 4.1 million tons annually or 4.5% of the national carbon footprint.^35^ Efforts to decarbonise healthcare must involve systemic clinical changes that simultaneously improve health outcomes, where preventive and rehabilitative care can play crucial roles.^36^ Digital interventions that support lifestyle changes, such as reducing sedentary behaviour, could lower health care-related emissions. However, digital technologies themselves contribute to GHG emissions through data centres, network infrastructure and device production, which must be considered when evaluating net climate benefits.^37^

Despite the recognised health risks of sedentary behaviour and its prevalence in patients with cancer, interventions specifically targeting sedentary behaviour reduction during cancer treatment remain scarce. Moreover, the effects of reducing sedentary behaviour on both short- and long-term health outcomes, as well as potential health economic and environmental benefits, have been insufficiently studied. To evaluate the effectiveness of such interventions, randomised controlled trials (RCTs) are needed to determine their effects on sedentary behaviour and related outcomes. However, effectiveness outcomes alone provide limited insight into broader health, economic and environmental implications. Combining quantitative and qualitative data on participants’ engagement with, use of, and perceived helpfulness of specific intervention components, together with the perspectives of healthcare professionals, may provide essential insights into intervention mechanisms and implementation in clinical practice.

This study aims to address these gaps in effectiveness, implementation and sustainability by investigating the effects of a novel digital support intervention to RedSedCan. While RCTs are essential for establishing causal effects on sedentary behaviour and health-related outcomes, they provide limited insight into how and why behaviour change occurs and how interventions can be implemented in clinical practice. Therefore, this study includes both quantitative and qualitative methods, in which the qualitative methods complement quantitative data by exploring the experiences of participants and the perspectives of healthcare professionals, thereby enabling a deeper understanding of behavioural processes and contextual factors. In addition, by including health economic and environmental evaluations, this study aims to generate knowledge about the broader sustainability implications of reducing sedentary behaviour, supporting future implementation and transferability in healthcare systems.

## Method

### Aims

The primary aim of the RedSedCan study is to evaluate the effects of a digital support intervention (the RedSedCan intervention) on sedentary time in individuals undergoing neoadjuvant or adjuvant treatment for breast, prostate or colorectal cancer.

The secondary aims are to examine the health effects of the intervention and its effect on physical activity, and to assess the potential health economic and environmental implications associated with its delivery. Additionally, this study aims to explore participants’ perceptions and experiences of the intervention as well as healthcare professionals’ attitudes, perceived barriers and facilitators related to supporting individuals with cancer in reducing sedentary behaviour.

Aims of the effect evaluation:

- To evaluate the effects of a 3-month digital support intervention on sedentary time (minutes/day).
- To evaluate the effects of a 3-month digital support intervention on sedentary bouts, perceived health-related quality of life, cancer-related fatigue and changes in health at 3, 6, 12 and 24-month follow-up assessments.
- To evaluate the effects of a 3-month digital support intervention on sedentary time, sedentary bouts, perceived health-related quality of life, cancer-related fatigue and changes in health at 6, 12 and 24-month follow-up assessments.
- To evaluate the health economic effects (ie, short- and long-term resource utilisation and social costs and benefits) of a digital support intervention.
- To calculate the impact of a digital support intervention on GHG emissions, directly through changes in behaviour and indirectly through healthcare, and the economic effects of these emissions.
- To explore patients’ perceptions and experiences of a digital support intervention.
- To explore healthcare professionals’ attitudes and perceived barriers and facilitating factors for supporting individuals with cancer to reduce sedentary behaviour and implement a digital support intervention for reducing sedentary behaviour among individuals with cancer.

Aims for sustainability and implementation evaluation:

- To evaluate the health economic effects (ie, short- and long-term resource utilisation and social costs and benefits) of a digital support intervention.
- To calculate the impact of a digital support intervention on GHG emissions, directly through changes in behaviour and indirectly through healthcare, and the economic effects of these emissions.
- To explore patients’ perceptions and experiences of a digital support intervention.
- To explore healthcare professionals’ attitudes and perceived barriers and facilitating factors for supporting individuals with cancer to reduce sedentary behaviour and implement a digital support intervention for reducing sedentary behaviour among individuals with cancer.

### Design

The RedSedCan study is a multicentre RCT with short- and long-term follow-ups, conducted alongside qualitative interview studies with study participants and healthcare professionals. The RCT is a two-armed trial, comprising an intervention group and a control group (figure 1), and will follow the Standard Protocol Items: Recommendations for Interventional Trials (SPIRIT) guidelines.^38^ In addition, two qualitative interview studies will be conducted to complement the RCT by exploring the perspectives of the participants and healthcare professionals on the intervention (figure 1). The study commenced on 1 March 2025, and the final follow-up assessments are expected to be completed by the end of 2029.

**Figure 1:**
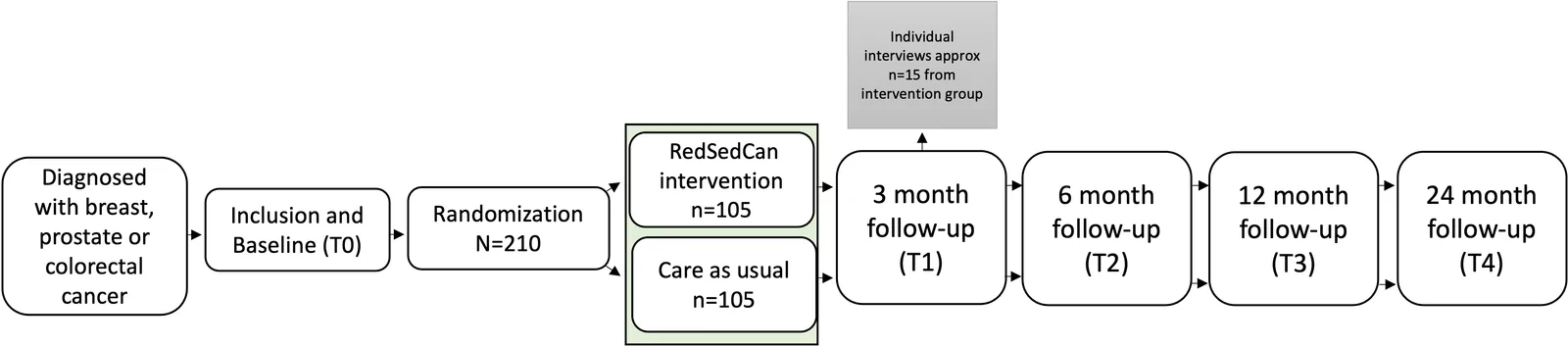
RedSedCan study design. RedSedCan, Reduce Sedentary behaviour during Cancer treatment.

### Setting

The RedSedCan study is a Swedish study including participants recruited from oncology departments at two regional hospitals in central Sweden.

### Participants

Individuals eligible to participate in the RCT are patients over 18 years of age who are diagnosed with breast, prostate or colorectal cancer and who are planning to undergo neoadjuvant or adjuvant treatment (chemotherapy, radiation, hormonal or targeted biological therapies). Individuals who are not fluent in Swedish, who are not able to walk independently (with or without assistive devices such as walkers) or who do not have access to a smartphone will be excluded. Additionally, individuals with dementia, severe psychiatric disease or functional limitations that prevent participation in the study procedures will also be excluded.

To address Aim V, a purposive sample of participants randomised to the intervention will be invited to participate in individual interviews at the end of the intervention period. In addition, to address Aim VI, a purposeful sample of healthcare professionals (eg, registered nurses, physicians or physiotherapists) working in cancer units will be recruited for focus group interviews. Purposive sampling will be used to ensure variation in cancer diagnosis, type of treatment and level of engagement in the intervention among RCT participants, as well as variation across clinical settings, professional roles and experience among healthcare professionals.

### Sample size

A total of 210 participants will be included in the RCT based on a power calculation for the primary outcome sedentary time (minutes/day) and anticipated dropout rate of 30%. The sample size calculation was based on data from a previous RCT in breast cancer survivors.^39^ The assumed between-group difference in sedentary time was 36.6 min/day, corresponding to a Cohen’s d of 0.467. Assuming a two-sided significance level of 0.05 and 80% statistical power, the required sample size was estimated at approximately 73 participants per group, corresponding to 146 participants in total. Allowing a dropout rate of 30%, the total sample size was increased to approximately 209 participants. To allow for equal allocation between the two groups, the final total sample size was rounded up to 210 participants (105 participants per group).

Approximately 10–15 participants from the intervention group will be interviewed to explore their experiences of reviving the digital support intervention. Recruitment of participants will continue until no new information emerges from the last three interviews. For the interviews with healthcare professionals, the final number of participants will be guided by availability; however, approximately 20 participants (eg, five individuals in four focus groups) are estimated to be sufficient.

### Recruitment and procedures

Participants in the RCT will be consecutively recruited from the oncology departments at two regional hospitals in central Sweden, starting in March 2025. Potential participants will be identified through multidisciplinary healthcare conferences or referral systems. At the start of their treatment, patients who meet the inclusion criteria and none of the exclusion criteria will receive brief oral information about the study from healthcare personnel, be provided with written study information and be asked for permission to be contacted by a member of the research team. Patients who are deemed eligible but not informed at the hospital will receive written study information by mail or email. A member of the research team will subsequently contact potential participants by telephone to provide additional information about the study, answer questions, confirm eligibility according to the study criteria and ask whether they wish to participate.

Following verbal consent, written informed consent will be obtained electronically via the Research Electronic Data Capture (REDCap) system.^40^ REDCap will also be used to generate a study-specific identification code for each participant, conduct randomisation, distribute surveys and send reminders (up to three per distribution point). In addition, REDCap will support intervention delivery by managing invitations and protocols for participant booking and goal setting. Participants who are unwilling or unable to use electronic consent forms and surveys will be provided with paper versions sent by mail, which they will return using a prepaid return envelope.

Participants will be evaluated at the following five measurements time (T) points: at baseline directly after enrolling in the RCT (T0) and at 3 (T1), 6 (T2), 12 (T3) and 24 (T4) months after baseline (figure 1). After completion of the baseline measurement, participants will be randomised at a 1:1 ratio using a computer-generated permuted-block randomisation scheme in REDCap, allocating participants to either the intervention (see description of the RedSedCan intervention) or the control group. Participants assigned to the control group will receive the standard care provided at their hospital (ie, care as usual). The random allocation sequence was generated by an independent statistician and implemented in REDCap. The allocation sequence will remain concealed from members of the research team responsible for participant enrolment and randomisation throughout the trial.

Potential participants for the interview study (Aim V) will be contacted by telephone after completion of the intervention and provided with written study information by mail or email. healthcare professionals (Aim VI) will be identified and recruited through managers at oncology units providing care for patients with cancer at regional hospitals in central Sweden, either by asking managers to identify eligible participants or by distributing study information to staff. Written informed consent will be obtained prior to participation in individual or focus group interviews.

### Data collection

Sedentary time (minutes/day), the primary endpoint, as well as sedentary bouts and physical activity as secondary outcomes, will be assessed using an Axivity AX3 accelerometer (AX3 Axivity, York, UK)^41 42^ attached to the thigh and worn continuously for 7 consecutive days. Raw acceleration data will be recorded at a sampling frequency of 25 Hz with a dynamic range of ±8 g. Data will be processed using the ActiPASS software, which currently applies the validated Acti4 algorithm to classify body postures and activities based on acceleration signals and thigh inclination relative to gravity.^43 44^ Based on criteria proposed by,^45^ a valid measured day should include a minimum of 20 hours of wear time, at least one waking period and one sleep interval. Patient-reported outcomes, including health-related quality of life, symptoms, perceived change in health, and self-efficacy to reduce sedentary behaviour, will be measured using validated instruments and study-specific questions at all measurement points (table 1). Health-related quality of life and symptoms will be measured with the European Organisation for Research and Treatment of Cancer Quality of Life Questionnaire^46^ and diagnose-specific modules.^47–49^ Cancer-related fatigue will be measured with the Brief Fatigue Inventory.^50^ Demographic and medical information will be collected through study-specific questions, medical records, and national quality registers. Treatment outcomes, that is, treatment completion and side effects, will be obtained from medical records. In addition, information on adverse events and on standard care received by participants, including, for example, physiotherapy and occupational therapy, will be gathered from medical records. The health economic evaluation (ie, loss of productivity, healthcare utilisation and cost of intervention) will be based on the following information: data from medical records; national quality registers for breast, prostate and colorectal cancer; Statistics Sweden; the internal accounting system within the healthcare organisation; and study-specific questions. Health-related quality of life will also be measured with the EuroQoL five-Dimensional Questionnaire,^51^ which will be used to calculate quality-adjusted life-years (QALYs). Selected and adapted questions from the Work Ability Index will be used to assess work ability.^52^ healthcare utilisation data will also be used to estimate GHG emissions. Changes in climate-related behaviours (eg, use of carbon-based transport vs active transport) will be measured using study-specific questions. The digital support intervention will also be evaluated using self-reported questions addressing the use, perceived utility, acceptance and appropriateness of the intervention components.

**Table 1:** Study variables and measurements

Outcome	Measurement	Measurement point
Sedentary behaviour Time spent sedentary Sedentary bouts	Accelerometer and study-specific activity diary	T0–T4
Physical activity Time spent physically active Physical activity level	Accelerometer and study-specific activity diary	T0–T4
Health-related quality of life	European Organisation for Research and Treatment of Cancer Quality of Life Questionnaire^46^ and diagnose-specific modules: breast cancer-specific module,^47^ prostate cancer-specific module (PR25),^48^ and colorectal cancer-specific module (CR29)^49^	T0–T4
Quality-adjusted life-years	EQ5D^51^	T0–T4
Cancer-related fatigue	Brief Fatigue Inventory^50^	T0–T4
Fear of movement	Tampa Scale for Kinesiophobia^68^	T0–T4
Self-perceived health	Patient Global Impression of Change health^69^	T0–T4
Self-efficacy for reducing sedentary behaviour	Study-specific question	T0–T4
Work ability	Work Ability Index^52^	T0–T4
Loss of productivity	Sick leave Study-specific questions	T0–T4
healthcare costs	National registers	T0–T4
Cost of intervention	Study-specific questions (cost of application use and purchase of new equipment for physical activity)	T0–T4
Outpatient visits	National registers Study-specific questions	T0–T4
Hospitalisation	National registers	T0–T4
Prescribed medication use	National registers	T0–T4
Active transportation	Study-specific questions	T0–T4
Diagnosis and treatment variables Treatment completion (dose adjustments, treatment delays and early discontinuation) Side effects	Medical records and quality registers	T0–T4
Demographic variables	Study-specific questions (age, sex, education level, employment status, occupation, income and comorbidities)	T0–T4
Overall digital use	Study-specific questions	T0–T4
Support in reducing sedentary behaviour	Study-specific questions	T0–T4
Evaluation of digital support Use Acceptability Appropriateness Goal fulfilment	Study-specific questions (intervention group only) and goal fulfilment ratings from meetings with physiotherapist	T1

EQ-5D, EuroQoL five-Dimensional.

In addition, qualitative data will be collected through individual interviews with participants randomised to the intervention as well as focus group interviews with healthcare professionals. All the interviews will be audio recorded and transcribed verbatim.

### The RedSedCan intervention

The digital support intervention (the RedSedCan intervention) was designed to reduce sedentary behaviour and involves a 12-week digital support programme consisting of several BCTs^53^ (table 2). The intervention was developed in several steps, beginning with an exploration of the target population’s experiences and opinions regarding sedentary behaviour and ways to support a non-sedentary lifestyle during neoadjuvant or adjuvant cancer treatment.^19 20^ These insights informed a participatory design study involving individuals from the target population and researchers, during which the intervention components were refined through discussions, testing, and quality evaluation of commercially available technology (eg, smartphone applications) (Henriksson *et al*, unpublished manuscript). The intervention was further assessed for feasibility and perceived usefulness in a single-case study (n=8) (Henriksson *et al*, unpublished manuscript).

**Table 2:** Intervention behaviour change techniques

Intervention component	Form (delivery mode and dose)	Behaviour change technique(s)*
Structured individual meetings with physiotherapists	Video/phone sessions; 4 meetings (intro+weeks 2, 8 and 12); 5–45 min; delivered by physiotherapist with oncology and behaviour change expertise	Goal setting (behaviour) Action planning Review behaviour goal(s) Feedback on behaviour Social support (emotional) Behaviour substitution Problem solving
Study-specific webpage	Short videos+text with information on sedentary behaviour and how to reduce sedentary behaviour during cancer treatment: accessible continuously during and after the 12-week intervention, reviewed between meetings	Information about health consequences Instruction on how to perform the behaviour Credible source
Smartphone step-counting application or equivalent	Steps App or equivalent: free or paid version (study covers paid version up to 1 year; recommended to use daily for self-monitoring and feedback)	Goal setting (behaviour) Feedback on behaviour Prompts/cues Self-monitoring of behaviour Review behaviour goals
Reminder/alarm function	Participant-configured smartphone alerts (eg, every 60 min during waking hours) to interrupt prolonged sitting: recommended to use daily or when convenient for the participant	Prompts/cues
Encouragement to engage in social support	Discussed at introduction; revisited as needed; involves family, friends and colleagues	Social support Restructuring the social environment
SMART goal setting	Set at introduction; refined at weeks 2, 8 and 12	Goal setting Action planning Implementation intentions
Maintenance planning (final meeting)	Week 12 during last meeting; establish maintenance goals and plan ongoing independent use of digital tools	Review outcome goals Action planning

* Behaviour change techniques as described by Michie *et al* in Behaviour Change Techniques Taxonomy v1. SMART: specific, measurable, achievable, relevant and time bound.

The RedSedCan intervention is person centred and delivered entirely through digital platforms. The intervention consists of four structured individual meetings with physiotherapists, access to a study-specific webpage with educational material, a step-counting mobile application (StepsApp or equivalent), and the use of smartphone reminder or alarm functions. Together, these components incorporate key BCTs, including goal setting, action planning, feedback, instruction, prompts/cues, problem solving and social support (table 2).

Participants will attend one introduction meeting and three follow-up meetings at 2, 8 and 12 weeks. Meetings will be conducted via video (eg, Zoom) or phone and scheduled at a time convenient for the participant, with physiotherapists (project staff) experienced in working with patients with cancer, behaviour change, sedentary behaviour and physical activity. During these meetings, the physiotherapist will discuss the participant’s current sedentary behaviour, physical activity, and preferences for activities to replace sedentary time, considering overall well-being, treatment-related challenges and individual life circumstances.

#### Introduction meeting

During the introductory meeting, the physiotherapist will explain the purpose and structure of the intervention and provide the participant with an opportunity to ask questions. Factors that may impact sedentary behaviour and physical activity, such as work or retirement status, available social support (eg, support from family, friends and colleagues) and perceived physical and psychological health during treatment, will be addressed. Participants will also be encouraged to seek support from their personal networks to reduce their sedentary time. The participants will also be asked whether they have visited the study-specific website; if not, they will be encouraged to review it before the next meeting.

The study-specific webpage offers short videos and written materials providing educational content and tips on sedentary behaviour, symptoms, treatment-related side effects during cancer treatment, the importance of reducing sedentary behaviour and strategies for reducing sedentary behaviour.

#### Digital tools (mobile app and reminder)

During the introduction meeting, the physiotherapist will ensure that the participant has downloaded a step-counting mobile application and understands how to use both the app and the reminder or alarm functions on their smartphone. If the participant already uses another health application or device that contains corresponding BCTs (table 2), they will be encouraged to continue using it. The step-counting application can be used either in a free version (which does not require participant registration) or in a paid version (which requires registration). Participants may choose which version they prefer to use. The study covers the cost of the paid version for use up to 1 year.

#### Goal setting (SMART)

At the end of the introductory meeting, the physiotherapist will support the participants in formulating clearly defined and actionable goals targeting sedentary behaviour and physical activity in accordance with the SMART framework, ensuring that the goals are Specific, Measurable, Achievable, Relevant and Time bound.^54^

#### Follow-up meetings

During each follow-up meeting, the physiotherapist will review the participant’s goals together with the participant, who rates goal attainment on a scale from 0 to 10. If the goals prove too easy or difficult to achieve, adjustments will be made, and goals can be replaced or added. In addition, the physiotherapist will follow up on the participants’ use of the application, the reminder function and the website. During the last follow-up (at 12 weeks), goals for maintenance will be set, and participants will be encouraged to continue using the intervention independently, without further meetings with the physiotherapist.

#### Intervention fidelity

Throughout the delivery of the intervention, the research team will hold weekly meetings to discuss and calibrate the delivery of the intervention and its components to ensure consistency and fidelity.

### Analysis

This is a multimethod study involving both quantitative and qualitative data. The primary analysis of the RCT will follow the intention-to-treat principle, with participants analysed according to their randomised allocation. Personnel involved in outcome assessment and statistical analysis are blinded to group allocation. The primary (sedentary time at baseline and 3 months) and secondary endpoints will be analysed using appropriate regression models, including multiple linear regression, mixed-model regression, analysis of covariance or multiple logistic regression. All models will be adjusted for baseline sedentary time, diagnosis, type of treatment and age.

Analyses of secondary outcomes and treatment effects at 6, 12 and 24 months will be considered secondary and exploratory, and p values and CIs will not be interpreted as confirmatory evidence unless an appropriate multiplicity adjustment is applied. Sensitivity analyses will include complete-case and per-protocol analyses. Missing data will be handled using multiple imputation if the missing-at-random assumption is considered appropriate.^55^

The health-economic evaluation will be conducted from a societal perspective over the 24-month follow-up period. Cost related to the intervention, healthcare utilisation and other relevant societal costs will be included in the analysis. Cost utility will be assessed based on incremental costs and QALYs (ie, a measure where one QALY equals 1 year in full health) for the intervention group compared with the control group. Cost and health outcomes occurring beyond the first year will be discounted in accordance with applicable Swedish guidelines.^56^

The environmental analysis will estimate GHG emissions associated with the RedSedCan intervention from a life-cycle perspective. The system boundaries will include emissions associated with the digital technology used to deliver the intervention, changes in participants’ climate-related behaviours and healthcare utilisation. Emissions from healthcare will be estimated based on the United Kingdom National Health Service tariffs,^57^ except for the emission of hospital energy consumption, which will be based on energy costs and official statistics from the Swedish Energy Agency.^58^ Differences in GHG emissions between the intervention and control groups will be estimated, and the corresponding economic value of emissions will be calculated using Swedish carbon dioxide tax rates.^59^

To identify which components of the digital support intervention are most central to the intervention (ie, core components), quantitative data on participants’ engagement with and use of specific intervention components, including perceived helpfulness, will be analysed descriptively as part of the process evaluation. In addition, exploratory analyses will examine associations between levels or patterns of engagement and changes in primary and secondary outcomes, as well as differences across participant subgroups defined by clinical and demographic characteristics.

Interviews with participants and healthcare professionals will be audio recorded, transcribed verbatim and analysed with inductive qualitative content analysis.^60^

### Patient and public involvement

Patients contributed to the design of the intervention through interview studies and a participatory design study involving discussions, testing and evaluation of commercially available digital technologies. Patients will not be involved in the recruitment or conduct of the RCT. They were also not involved in the development of the trial outcome measures, which were selected based on the study aims and existing evidence. The study results will be disseminated to participants in an accessible format and shared with relevant patient communities through appropriate channels.

## Ethics and dissemination

The planning and reporting of this study are in accordance with the Helsinki Declaration and later amendments.^61^ The project has received ethical approval from the Swedish Ethical Review Authority (Ref no. 2024-04632-01).

All participants will be asked to provide written informed consent before enrolling in the different parts of the RedSedCan study, either digitally through a web-based link or if preferred, in paper format (online supplemental material 1). The written and oral information given to all potential participants includes details about the study, what participation entails, how the data will be safeguarded, how the data are anonymised and analysed only at the group level, and how the participants can later access the results. It is also emphasised that participation is voluntary and may be withdrawn at any time without giving a reason (online supplemental material 1).

Physical activity, even at high intensity, is considered safe to perform during oncological treatment.^1 62^ The intervention aims to reduce sedentary behaviour by replacing sedentary behaviour with any chosen intensity of activity, which explains why the risk of exercise-related injuries in this intervention is considered low. Given that physical activity has several benefits during neoadjuvant or adjuvant cancer treatment, asking participants to refrain from physical activity if they are randomised to the control condition is inappropriate. Instead, they will receive care as usual, which may include encouragement and support to be physically active from healthcare professionals.

Researchers will have access to data in the RedSedCan study. The data will not be openly accessible because of the General Data Protection Regulation (2016/679). Pseudonymised data may be shared with other researchers only following approval from the Swedish Ethical Review Authority and in accordance with applicable legislation and the scope of the approved ethical permission. Individual participant data will not be made publicly available.

The findings from this study will be disseminated through publication in peer-reviewed journals and presentations at national and international scientific conferences.

## Discussion

Approximately one in three individuals in Sweden is estimated to receive a cancer diagnosis during their lifetime,^63^ and owing to advances in cancer detection and treatment, an increasing number of individuals are living with cancer.^64^ Consequently, identifying strategies that can reduce treatment-related side effects and support recovery and long-term health is of growing importance.^65^ Sedentary behaviour often increases during cancer treatment because of fatigue, pain, psychological distress and other treatment-related symptoms.^6^ Interventions that support individuals with cancer in maintaining movement in everyday life during treatment may therefore contribute to improved health outcomes both in the short and long term.

The RedSedCan study applies a novel approach by targeting sedentary behaviour as the primary outcome. While previous research has emphasised the importance of increasing physical activity during and after treatment, few interventions have specifically aimed to reduce sedentary time and its relationship to adverse health outcomes. By encouraging participants in the RCT to both interrupt prolonged sedentary bouts and to replace time spent sedentary with activities of any chosen intensity, the RedSedCan intervention addresses an underexplored but clinically relevant behaviour with the potential to improve both short- and long-term health during and after cancer treatment.

In addition to the main outcome of sedentary behaviour, we will investigate the effects of the RedSedCan intervention on health-related quality of life and other health outcomes, such as cancer-related fatigue. In addition, sustainability perspectives will be explored through cost-effectiveness and the impact on GHG emissions associated with healthcare delivery and lifestyle behaviours. By integrating social, economic and environmental sustainability perspectives, the RedSedCan study aims to contribute to the growing field of sustainable healthcare and provide knowledge about the potential societal impact of digital lifestyle interventions in oncological care, allowing for a more comprehensive understanding of the overall value of the intervention.

A key strength of the RedSedCan intervention is that it was developed together with patients undergoing cancer treatment, which is in accordance with the recommendations of the Medical Research Council framework for complex interventions.^27^ The intervention was developed through several preparatory studies, including qualitative research exploring patients’ experiences of sedentary behaviour during neoadjuvant or adjuvant cancer treatment and their preferences regarding support for reducing sedentary time. These studies highlight that sedentary behaviour during treatment is often perceived as an adaptation to treatment-related side effects and that patients express a need for individual support from healthcare professionals.^19 20^ Insights from these qualitative studies informed the development of the digital support intervention through a co-production process involving individuals from the target population. By incorporating patient perspectives during the development phase, the intervention was designed to be relevant, feasible and acceptable for individuals undergoing cancer treatment. The RCT described in this protocol represents the evaluation phase of the intervention development process.^27^

In addition to assessing the effectiveness of the intervention in reducing sedentary behaviour, the RedSedCan study includes an evaluation aimed at exploring participants’ experiences with the digital intervention. These insights are expected to provide important contextual information for interpreting trial outcomes and understanding potential mechanisms of behavioural change. Healthcare professionals play important roles in promoting behavioural change among patients and in facilitating the integration of digital health interventions into clinical practice. By exploring perceived barriers and facilitating factors related to supporting reductions in sedentary behaviour and implementing digital interventions within oncological care, this study aims to generate knowledge about organisational and contextual factors that may influence future implementation.

The RedSedCan intervention systematically applies evidence-based BCT, including goal setting, action planning, health information, self-monitoring, feedback and social support.^53^ The BCTs used in the study aim to support both the initiation and maintenance of behaviour change throughout the intervention period. The combination of structured meetings with physiotherapists and digital tools supporting self-monitoring and reminders enables participants to receive both professional guidance and continuous behavioural feedback. Goal setting based on the SMART framework supports participants in establishing realistic and individualised goals that can be adjusted throughout the intervention period. By combining these BCTs with person-centred discussions, the RedSedCan intervention aims to support both the initiation and maintenance of behavioural change. Another key component of the intervention is the use of digital support tools. Digital solutions may provide accessible and scalable approaches to support behaviour change, particularly for individuals undergoing cancer treatment who may experience limited energy levels or difficulties attending in-person interventions.

The digital format enables flexible participation and reduces the need for travel to healthcare facilities. In addition, commonly available technologies, such as smartphone applications and reminder functions, can support self-monitoring and prompt regular movement breaks throughout the day. By combining digital support with professional guidance from physiotherapists, the intervention aims to integrate behaviour change support into participants’ everyday environments.

### Strengths and limitations

The RedSedCan study has several methodological strengths. First, the RCT design provides a robust framework for evaluating the effectiveness of the intervention. Second, sedentary time, the primary endpoint, is measured objectively using a thigh-worn accelerometer, reducing the risk of self-report bias associated with self-reported measures. Third, the multidisciplinary research team comprises both researchers and clinicians with complementary expertise in oncology, physical activity, behavioural science, public health and implementation research. The team includes members actively involved in related research on physical activity, sedentary behaviour, and cancer, and it spans a range of academic seniority, including senior researchers, postdoctoral fellows and early-career researchers. This combination of expertise strengthens the scientific robustness and relevance of the study and supports a clearly defined intervention and consistent delivery across sites. Standardised introductory meetings and follow-ups, as well as weekly research group meetings, will enhance intervention fidelity.^27^

However, some potential limitations should be acknowledged. As the intervention involves active participant engagement and support from the research team, participants and the research team delivering the intervention cannot be blinded to treatment allocation. Recruitment to intervention studies is generally challenging, which may also affect the representativeness of the study sample. Additionally, being dependent on time-pressured clinical staff for recruitment may limit the likelihood of all patients being invited to participate. Participation in the study requires access to a smartphone and a certain level of digital skills, which may limit participation among some individuals and potentially affect the generalisability of the findings. However, Sweden is among the most digitalised countries in the world, with 67% of the population aged 16–74 years estimated to have at least basic digital skills.^66^ Attrition may occur because of treatment side effects or disease progression during the study period. The issue of participants who stop using support or are lost to follow-up is common in mHealth interventions.^67^ Nevertheless, the various strategies, such as digital/phone-only meetings, flexible scheduling and the use of reminders, implemented to support participant engagement and reduce attrition should be acknowledged.

## Supplementary material

10.1136/bmjopen-2026-125120 online supplemental file 1
