# Supplementary material for: Does a digital support intervention reduce sedentary behaviour during cancer treatment in individuals with breast, prostate or colorectal cancer? Study protocol for a Swedish multicentre randomised controlled trial
Source: BMJ Open. 2026 Oct 29;16(10):e125120. doi: 10.1136/bmjopen-2026-125120 (PMC13723854; doi:10.1136/bmjopen-2026-125120)
Supplement: Supplementary file 1 [file bmjopen-16-10-s001.pdf]

English translation of information sheet to potential research participants

Reducing Sedentary Behaviour During Cancer Treatment – The RedSedCan Study

Version 260223

Information for Research Participants

We would like to ask you if you would like to participate in a research project. This document provides information about the project and what participation entails.

What is the project about and why are you being asked to participate?

Previous studies have shown that sedentary behaviour often increases among people undergoing cancer treatment and that sedentary behaviour in general is associated with an increased risk of ill health. Sedentary behaviour refers to the time a person spends sitting or lying down while awake. The purpose of this project is therefore to investigate how people undergoing cancer treatment can prevent an increase in sedentary behaviour or reduce their sedentary behaviour by replacing sedentary time with daily activities or physical activity.

You are being asked to participate because you are about to start or have already started cancer treatment. You can participate regardless of your previous or current level of physical activity or treatment plan. We have obtained your information from one of the clinics participating in the project.

To participate in the project, you need to be able to speak and understand Swedish and be able to stand and walk. You also need access to a mobile phone (iPhone or Android) with an internet connection, as the interventions being tested in the project are delivered digitally (see "How does the project work?" below).

Mälardalen University is the research principal for the project. The research principal is the organisation responsible for the study. The research project has been approved by the Swedish Ethical Review Authority (reference number 2024-04632-01).

How does the project work?

If you choose to participate in the project, you will be randomly assigned to one of two groups:

Group 1: A group receiving digital support

If you are randomly assigned to Group 1, you will test a digital support programme for 3 months consisting of the following components:

a) A step-counter app that is downloaded to your mobile phone in connection with an introductory meeting.

b) Your mobile phone's reminder function (alarm) will be used to set times for, for example, movement breaks or other activities.

c) A website containing information in the form of texts, videos and links about sedentary behaviour and tips on how you can reduce your sedentary behaviour.

d) Introductory meeting: Before you start using the programme, you will receive an introduction from project staff who are physiotherapists. They will explain how to use the programme. The introduction will take place digitally via telephone or video call. You will receive help setting goals tailored to your needs for reducing sedentary behaviour.

e) Follow-up meetings: You will meet with the project staff on three occasions (after 2, 8 and 12 weeks) to follow up on how things are going and receive support and assistance in reducing your sedentary behaviour. These meetings will also take place digitally. You will also be asked questions about the extent to which you have achieved your goals for reducing sedentary behaviour.

You may also be informed about and invited to participate in an interview study focusing on your experience of using the digital support.

Group 2: Control group

If you are randomly assigned to Group 2, you will only be followed up by completing questionnaires on several occasions and wearing a movement monitor (see information below).

Measurement occasions

All participants (Groups 1 and 2) will be assessed before randomisation and after 3 months, 6 months, 12 months and 24 months. Before each measurement occasion, you will receive a reminder that the measurement is approaching, along with information about how it will be carried out.

Movement measurement: At each measurement occasion, you will wear a movement monitor that measures sedentary behaviour and physical activity. The movement monitor is worn for 7 days, and during this period you will also record the activities you have performed and how long you sleep each night in a diary. The movement monitor is attached to your thigh using skin-friendly tape. Before each measurement occasion, the movement monitor will either be handed

to you by project staff or mailed to your home. Once the measurement period is complete, you will return the monitor by post using a prepaid envelope.

Questionnaires: At each measurement occasion, you will complete questionnaires. The questions you answer will vary between measurement occasions. The questionnaires will be completed either digitally or on paper. The questionnaires contain questions about your living situation, health and finances. You will also answer questions about sedentary behaviour, use of apps and daily transportation.

We may also obtain information about your diagnosis and treatment from healthcare medical records, as well as information from healthcare quality registers, the National Board of Health and Welfare (Socialstyrelsen), and the Swedish Social Insurance Agency (Försäkringskassan).

Possible consequences and risks of participating in the project

Participation in the project involves no apparent risks.

Previous research has shown that sedentary behaviour in general is associated with an increased risk of ill health. Previous research has also shown that physical activity during cancer treatment is safe to perform and may reduce certain side effects, such as cancer-related fatigue.

What will happen to my personal data?

The project will collect and register information about you. All responses from questionnaires, data from the movement monitor, and data from medical records and registers will be coded.

No unauthorised person will have access to your responses, and the results will be reported at group level. The data will be stored for 10 years.

Mälardalen University is responsible for processing your personal data. Under the EU General Data Protection Regulation (GDPR), you have the right to access, free of charge, the information about you that is processed in the project and, if necessary, to have any errors corrected. You can also request that information about you be erased and that the processing of your personal data be restricted. However, the right to erasure and restriction of processing does not apply when the data are necessary for the research in question.

If you wish to access the information, please contact the principal investigator, Petra von Heideken Wågert, telephone: 021-10 31 88.

The Data Protection Officer at Mälardalen University can be contacted by email at: dso@mdu.se.

If you are dissatisfied with how your personal data are processed, you have the right to lodge a complaint with the Swedish Authority for Privacy Protection (Integritetsskyddsmyndigheten), which is the supervisory authority.

How will I receive information about the results of the project?

The results of the study will be presented at scientific conferences and published in scientific journals.

Insurance and compensation

The patient insurance and your private insurance are valid during participation in the study. The project does not compensate for any loss of income or travel expenses incurred as a result of participation in the study.

Participation is voluntary

Participation in the project is voluntary, and you can choose to withdraw from participation at any time. If you choose not to participate or wish to withdraw from the study, you do not need to give a reason, and this will not affect your future care or treatment.

If you wish to withdraw from the study, please contact one of the researchers in the study (see contact details below).

Persons responsible for the project

Researchers in the project

Johanna Zetterlund
Physiotherapist, researcher
Mälardalen University
Telephone: 021-107091

Anna Henriksson
Nurse, researcher
Mälardalen University
Telephone: 021-101585

Lisa Rådman
Physiotherapist, researcher
Mälardalen University
Telephone: 016-153702

Principal Investigator

Petra von Heideken Wågert
Physiotherapist, Professor of Physiotherapy
Mälardalen University

Email address for the project and project group:
redsedcan@mdu.se

Consent to Participate in the RedSedCan Study

I have received oral and written information about the study and have had the opportunity to ask questions. I have been given a copy of the written information to keep

I consent to participate in the ***RedSedCan study***.

____

Place and date Signature

____

Name in block letters

Information till forskningspersoner

Vi vill fråga dig om du vill delta i ett forskningsprojekt. I det här dokumentet får du information om projektet och om vad det innebär att delta.

Vad handlar projektet om och varför vill ni att jag ska delta?

Syftet med detta projekt är att undersöka hur personer som genomgår behandling mot cancer kan minska (reducera) sitt stillasittande. Med stillasittande menas den tid som en person sitter eller ligger ner under den vakna tiden på dygnet.

Du blir tillfrågad om att delta eftersom du ska påbörja eller har påbörjat en behandling mot cancer. Vi har fått tillgång till dina uppgifter från en av de kliniker som ingår i projektet.

För att medverka i projektet behöver du kunna tala och förstå svenska samt kunna stå och gå. Du behöver även ha tillgång till en mobiltelefon (Iphone eller Android) med internetuppkoppling eftersom insatserna som testas i projektet levereras digitalt (se nedan under "Hur går projektet till?").

Forskningshuvudman för projektet är Mälardalens universitet. Med forskningshuvudman menas den organisation som är ansvarig för studien. Forskningsprojektet är godkänt av Etikprövningsmyndigheten (diarienummer 2024-04632-01).

Hur går projektet till?

Om du väljer att delta i projektet kommer du att lottas till en av två grupper:

1) En grupp som tar del av ett digitalt självhanteringsprogram

Om du lottas till grupp 1 innebär det att du under 3 månader kommer att få testa ett digitalt självhanteringsprogram som består av tre delar:

a) En stegräknarapp som laddas ner på din mobiltelefon i samband med en introduktionsträff.

b) Din mobiltelefons påminnelsefunktion (alarm) används för att ställa in tider för tex rörelsepauser eller andra aktiviteter.

c) En hemsida med information i form av texter, filmer och länkar om stillasittande och tips på hur du kan minska ditt stillasittande.

Introduktionsträff: innan du börjar använda programmet får du en introduktion av personal i projektet till hur du ska använda programmet. Introduktionen sker i samband med ett besök på sjukhuset. Du kommer att få hjälp att sätta mål som är anpassade efter dina behov för minskat stillasittande.

Uppföljningsträffar: du kommer att vid tre tillfällen (efter 2, 8 och 12 veckor) att träffa projektpersonalen igen för att följa upp hur det går och få stöd och hjälp att minska ditt stillasittande. Dessa träffar kan ske i samband med besök på sjukhus, digitalt (via tex videosamtal) eller via telefon. Du också att få svara på frågor om hur väl dina mål för minskat stillasittande uppfyllts.

Du kan även att komma att informeras och tillfrågas om du vill delta i en intervjustudie som handlar om hur du upplevt att använda självhanterings-programmet.

2) Kontrollgrupp

Om du lottas till grupp 2 kommer du enbart att följas upp genom att vid upprepade tillfällen fylla i enkäter och bära en rörelsemätare (se informationen nedan).

Mättillfällen

Alla personer (grupp 1 och 2) som deltar i projektet kommer att mätas innan lottningen, efter 3 månader, 6 månader, 12 månader och 24 månader. Inför varje mättillfälle kommer du att få en påminnelse om att det snart är dags och information om hur det kommer att gå till.

Rörelsemätning: vid varje mättillfälle kommer du att få bära en rörelsemätare som mäter stillasittande och fysisk aktivitet. Rörelsemätaren bärs under 7 dagar och du får samtidigt anteckna vilka aktiviteter du utfört samt hur länge du sover varje natt i en dagbok. Rörelsemätaren tejpas fast på låret med en hudvänlig tejp. Inför varje mättillfälle kommer rörelsemätaren antingen lämnas ut till dig från projektpersonal eller postas hem till dig. Efter mätningen är klar postar du tillbaka mätaren i ett förbetalt kuvert.

Frågeformulär: vid varje mättillfälle kommer du att få svara på frågeformulär. Vilka frågor som du svarar på skiljer sig åt mellan mättillfällena. Fråge-formulären fylls i antingen digitalt eller via pappersenkäter. Frågeformulären innehåller frågor om din livssituation, hälsa och ekonomi. Du får även svara på frågor om stillasittande, användning av appar samt frågor om dagliga transporter.

Vi kommer även att inhämta information om din diagnos och behandling från sjukvårdens journalsystem samt information från sjukvårdens kvalitetsregister, Socialstyrelsen och Försäkringskassan.

Möjliga följder och risker med att delta i projektet

Att delta i projektet innebär inga uppenbara risker. Att delta i projektet innebär inga uppenbara risker.

Tidigare forskning har visat att stillasittande generellt innebär ökad risk för ohälsa. Tidigare forskning har även visat att fysisk aktivitet under cancerbehandling är säkert att utföra och kan minska vissa biverkningar såsom cancerrelaterad trötthet.

Vad händer med mina uppgifter?

Projektet kommer att samla in och registrera information om dig. Alla svar från frågeformulär, data från rörelsemätaren och data från medicinsk journal och register kommer att kodas.

Ingen obehörig kommer att få ta del av dina svar och resultaten redovisas på gruppnivå. Data kommer att sparas i 10 år.

Ansvarig för dina personuppgifter är Mälardalens universitet. Enligt EU:s dataskyddsförordning har du rätt att kostnadsfritt få ta del av de uppgifter om dig som hanteras i projektet, och vid behov få eventuella fel rättade. Du kan också begära att uppgifter om dig raderas samt att behandlingen av dina personuppgifter begränsas. Rätten till radering och till begränsning av behandling av personuppgifter gäller dock inte när uppgifterna är nödvändiga för den aktuella forskningen. Om du vill ta del av uppgifterna ska du kontakta huvudansvarig forskare Petra von Heideken Wågert, telefonnummer: 021-10 31 88. Dataskyddsombud vid Mälardalens universitet nås på e-post: dso@mdu.se. Om du är missnöjd med hur dina personuppgifter behandlas har du rätt att ge in klagomål till Integritetsskyddsmyndigheten, som är tillsynsmyndighet.

Hur får jag information om resultatet av projektet?

Resultaten från studien kommer att redovisas vid vetenskapliga konferenser samt i vetenskapliga tidskrifter.

Försäkring och ersättning

Patientförsäkringen och din privata försäkring gäller vid deltagande i studien. Projektet ersätter inte eventuellt inkomstbortfall eller ersättning för resekostnader som uppstår till följd av deltagande i studien.

Deltagandet är frivilligt

Att delta i projektet är frivilligt och du kan när som helst välja att avbryta deltagandet. Om du väljer att inte delta eller vill avbryta ditt deltagande behöver du inte uppge varför, och det kommer inte heller att påverka din framtida vård eller behandling. Om du vill avbryta ditt deltagande ska du kontakta forskare i studien (se kontaktuppgifter nedan).

Ansvariga för projektet

Forskare i projektet:
Anna Henriksson,
Leg. sjuksköterska, Forskare, Mälardalens universitet
E-post: anna.henriksson@mdu.se

Huvudansvarig forskare:
Petra von Heideken Wågert
Professor i fysioterapi, leg. sjukgymnast
E-post: petra.heideken.wagert@mdu.se
