# Supplementary material for: Does a digital support intervention reduce sedentary behaviour during cancer treatment in individuals with breast, prostate or colorectal cancer? Study protocol for a Swedish multicentre randomised controlled trial
Source: BMJ Open. 2026 Oct 29;16(10):e125120. doi: 10.1136/bmjopen-2026-125120 (PMC13723854; doi:10.1136/bmjopen-2026-125120)
Supplement: Supplementary file 2 [file bmjopen-2026-125120.reviewer_comments.pdf]

PEER REVIEW HISTORY

BMJ Open publishes all reviews undertaken for accepted manuscripts. Reviewers are asked to complete a checklist review form and are provided with free text boxes to elaborate on their assessment. These free text comments are reproduced below.

ARTICLE DETAILS

Title (Provisional)

Does a Digital Support Intervention Reduce Sedentary Behavior during Cancer Treatment in individuals with Breast, Prostate or Colorectal Cancer? Study Protocol for a Swedish Multicenter Randomized Controlled Trial

Authors

Rådman, Lisa; Zetterlund, Johanna; von Heideken Wågert, Petra; Henriksson, Anna

---

VERSION 1 - REVIEW

**Reviewer**	**1**
**Name**	**Mulita, Francesk**
**Affiliation**	**University General Hospital of Patras, Department of Surgery, Breast Unit**
**Date**	**25-Jul-2026**
**COI**	**NA**

I would like to congratulate the authors on their fascinating work regarding this interesting report entitled "A Digital Support Intervention to Reduce Sedentary Behavior during Cancer Treatment in Individuals with Breast, Prostate or Colorectal Cancer: Study Protocol for the RedSedCan Study". The manuscript is well-written and the incorporated figures make the study easy to follow.

I strongly recommend acceptance for publication of the paper after minor revision.

1) I would like a brief discussion on Psychiatric disease in surgically treated colorectal cancer patients and consider citing the recently published article:

chrome-extension://efaidnbmnnnibpcajpcglclefindmkaj/https://www.surgchronicles.gr/components/com_journals/journals/2025/3/25.pdf

2) I would suggest a brief discussion on the role of deep learning in Colorectal Cancer (CRC) diagnosis. According to the literature, further investigation has been conducted into the potential clinical practice implementation of deep learning algorithms for the classification

and diagnosis of CRC histopathology images. The advancement made possible by deep learning algorithms has the potential to improve CRC detection's accuracy and efficacy.

Consider citing:

https://pubmed.ncbi.nlm.nih.gov/37937113/

https://pubmed.ncbi.nlm.nih.gov/38572457/

3) I want a brief discussion on Screening of healthcare staff for the prevention of colon cancer

https://pubmed.ncbi.nlm.nih.gov/41081069/

**Reviewer**	**2**
**Name**	**Giacomelli, Luca**
**Affiliation**	**Polistudium srl**
**Date**	**27-Jul-2026**
**COI**	**NA**

The protocol is well conceived, but several points warrant attention. The power calculation is the most pressing: reductions in sedentary time of 62% versus 45% are implausible as stated, and the assumed effect size, its source and the outcome metric should be restated in minutes per day. Please designate a single primary endpoint and timepoint, specify intention-to-treat as the primary analysis, and describe how multiplicity across four follow-ups and numerous secondary outcomes will be handled. Accelerometer processing requires detail: sampling frequency, epoch, non-wear and valid-day criteria, the algorithm used, and the operational definition of a sedentary bout. Stratification by site and diagnosis, blinded outcome assessment and data processing, and systematic recording of harms and of physical activity advice received by controls would strengthen internal validity. The health-economic and life-cycle analyses need perspective, time horizon, discounting and system boundaries. Several citations misalign (SPIRIT as reference 36; PGIC linked to the PG-SGA; PR26 versus PR25; a 2026 report dated October; a URL retaining a chatbot tracking parameter), and any AI assistance should be disclosed. Finally, remove anticipatory conclusions and supply a higher-resolution Figure 1.

VERSION 1 - AUTHOR RESPONSE

	Comment	In manuscript	Response
Reviewer 2	The power calculation is the most pressing: reductions in sedentary time of 62% versus 45% are implausible as stated, and the assumed effect size, its source and the outcome metric should be restated in minutes per day	Sample size	Thank you for highlighting this important issue. We agree that the original power calculation was insufficiently described and that the reported 62% versus 45% reductions were unclear for the continuous primary outcome. We therefore reassessed the sample size calculation using the same previous study by Lynch et al. (reference 38 in manuscript), but based the calculation on sedentary time expressed in minutes/day. The assumed between-group difference was 36.6 minutes/day, corresponding to a Cohen's *d* of 0.467. With a two-sided α of 0.05 and 80% power, the required sample was 73 participants per group. Allowing for 30% dropout resulted in a total sample of approximately 209 participants, which was rounded to 210 (105 per group). The revised power analysis was reviewed by a statistician. The manuscript has been revised accordingly. See page 8 Sample size
Reviewer 2	Please designate a single primary endpoint and timepoint	Aims/Analysis/Abstract	Thank you for this important comment. We have revised the manuscript to clearly designate a single primary endpoint and timepoint. The primary endpoint is now defined as objectively measured sedentary time (minutes/day), with the primary assessment at 3 months. The remaining outcomes and follow-up assessments are presented as secondary outcomes. We have also revised the wording throughout the manuscript to ensure consistency, including the study aims, the description of outcome measurements, the sample size section, and the "Strengths and limitations of this study" section.
Reviewer 2	Specify intention-to-treat as the primary analysis, and describe how multiplicity across four follow-ups and numerous secondary outcomes will be handled	Analysis	Thank you for this important comment. We have revised the Statistical Analysis section to specify intention-to-treat as the primary analysis, with participants analyzed according to their randomized allocation. The primary endpoint will be analyzed at the prespecified primary timepoint, while analyses of the additional follow-up assessments and secondary outcomes will be considered secondary. We have also added a prespecified strategy for handling multiplicity across follow-up timepoints and secondary outcomes. Complete-case and per-protocol analyses are now explicitly described as sensitivity analyses.
Reviewer 2	Accelerometer processing requires detail: sampling frequency, epoch, non-wear and valid-day criteria, the algorithm used, and the operational definition of a sedentary bout.	Data collection	We have clarified the accelerometer processing procedure, including sampling frequency (25 Hz, ±8 g), the use of the Acti4 algorithm via ActiPASS, and valid-day criteria. As activity classification is based on raw acceleration data, no predefined epoch length was applied.
Reviewer 2	Stratification by site	Data collection	Thank you for this valuable comment. We agree

	and diagnosis, blinded outcome assessment and data processing, and systematic recording of harms and of physical activity advice received by controls would strengthen internal validity.		that systematic recording of harms and of care received by participants is important for strengthening the internal validity of the study. We will systematically collect information on adverse events as well as standard care received during the study period, including physiotherapy and occupational therapy, from participants' medical records. This will allow us to account for relevant co-interventions and to describe any differences in care received between the intervention and control groups.<br><br>Regarding blinding, outcome assessment and data analysis will be performed by personnel blinded to treatment allocation. However, blinding of participants and intervention staff is not feasible because the nature of the intervention requires participants to be aware of the behavioural intervention aimed at reducing sedentary behaviour, and intervention staff need to be aware of participants' allocation in order to deliver the intervention. We have therefore ensured that the personnel involved in outcome assessment and statistical analysis are blinded to group allocation.<br><br>Randomisation will not be stratified by site or diagnosis. Instead, potential differences related to site and diagnosis will be accounted for in the statistical analyses. Site and diagnosis will be included as covariates in the prespecified statistical models. We have clarified these procedures in the method section.
Reviewer 2	The health-economic and life-cycle analyses need perspective, time horizon, discounting and system boundaries.	Analysis	Thank you for this comment. We have clarified the health-economic evaluation in the revised manuscript. The analysis will be conducted from a societal perspective over the 24-month follow-up period and will include intervention-related costs, health-care utilization, and other relevant societal costs. Cost-utility will be assessed based on incremental costs and quality-adjusted life years (QALYs), with QALYs calculated from EQ-5D data. Costs and health outcomes occurring beyond the first year will be discounted in accordance with applicable Swedish health-economic guidelines. We have also clarified the system boundaries for the life-cycle analysis and the estimation of greenhouse gas emissions.
Reviewer 2	Several citations misalign (SPIRIT as reference 36; PGIC linked to the PG-SGA; PR26 versus PR25; a 2026 report dated October; a URL retaining a chatbot tracking parameter), and any AI assistance should be disclosed.	References	Thank you for identifying these issues. We have carefully reviewed the reference list and in-text citations and corrected the misalignments, including the SPIRIT, PGIC/PG-SGA, and PR26/PR25 references (PR25 is the correct one). We have also corrected the publication date of the 2026 report and removed the chatbot tracking parameter from the URL. AI was used in some sections solely for language editing and improving the wording. The scientific content and conclusions are those of the authors.

Reviewer 2	Finally, remove anticipatory conclusions	Discussion/Conclusion	We have revised the manuscript to remove anticipatory conclusions
Reviewer 2	Supply a higher-resolution Figure 1	Figure 1	A higher resolution Figure 1 is provided as a separate file.
